# Chemoresistance to Valproate Treatment of Bovine Leukemia Virus-Infected Sheep; Identification of Improved HDAC Inhibitors

**DOI:** 10.3390/pathogens1020065

**Published:** 2012-10-08

**Authors:** Nicolas Gillet, Fabian Vandermeers, Alix de Brogniez, Arnaud Florins, Annamaria Nigro, Carole François, Amel-Baya Bouzar, Olivier Verlaeten, Eric Stern, Didier M. Lambert, Johan Wouters, Luc Willems

**Affiliations:** 1 Molecular and Cellular Epigenetics (GIGA) and Molecular Biology (GxABT), University of Liège, Liège 4000, Belgium; E-Mails: n.gillet@ulg.ac.be (N.G.); fabian.vandermeers@ulg.ac.be (F.V.); alix.debrogniez@doct.ulg.ac.be (A.B.); florinsarnaud@hotmail.com (A.F.); annamarianigro@hotmail.it (A.N.); francois-carole@hotmail.com (C.F.); amelbzr@yahoo.com (A.-B.B.); olivier.verlaeten@gmail.com (O.V.); 2 Pharmaceutic Chemistry and Radiopharmacy Unit, Louvain Drug Research Institute, University of Louvain, Brussels 1000, Belgium; E-Mails: dr.eric.stern@gmail.com (E.S.); didier.lambert@uclouvain.be (D.M.L.); 3 Biological Chemistry, Facultés Universitaires Notre-Dame de la Paix, Namur 5000, Belgium; E-Mail: johan.wouters@fundp.ac.be (J.W.)

**Keywords:** BLV, HDAC inhibitor, leukemia, HTLV

## Abstract

We previously proved that a histone deacetylase inhibitor (valproate, VPA) decreases the number of leukemic cells in bovine leukemia virus (BLV)-infected sheep. Here, we characterize the mechanisms initiated upon interruption of treatment. We observed that VPA treatment is followed by a decrease of the B cell counts and proviral loads (copies per blood volume). However, all sheep eventually relapsed after different periods of time and became refractory to further VPA treatment. Sheep remained persistently infected with BLV. B lymphocytes isolated throughout treatment and relapse were responsive to VPA-induced apoptosis in cell culture. B cell proliferation is only marginally affected by VPA *ex vivo*. Interestingly, in four out of five sheep, *ex vivo* viral expression was nearly undetectable at the time of relapse. In two sheep, a new tumoral clone arose, most likely revealing a selection process exerted by VPA *in vivo*. We conclude that the interruption of VPA treatment leads to the resurgence of the leukemia in BLV-infected sheep and hypothesize that resistance to further treatment might be due to the failure of viral expression induction. The development of more potent HDAC inhibitors and/or the combination with other compounds can overcome chemoresistance. These observations in the BLV model may be important for therapies against the related Human T-lymphotropic virus type 1.

## 1. Introduction

Bovine Leukemia Virus (BLV) is the causative agent of Enzootic Bovine Leukemia (EBL), which is characterized by an accumulation of transformed B-cells in the peripheral blood and/or by the onset of tumors in different tissues [1]. BLV infection can also be experimentally transmitted to sheep, a species in which pathogenesis is more acute [2]. A paradox of BLV pathogenesis is that the virus is apparently not expressed but, concomitantly, a strong immune response against BLV is present in infected animals [3,4]. We previously proposed a model that recapitulates these mechanisms [5]: we postulated that the virus is expressed in a fraction of provirus carrying cells, leading to their destruction by the host immune response. According to this model, silencing of viral expression is a prerequisite for cell persistence and accumulation. We hypothesized that induction of viral expression would deplete the silent pool leading to a reduction of the proviral loads. As a proof of concept, we provided evidence that valproate (VPA), an inhibitor of deacetylases, activates BLV gene expression in transient transfection experiments and in short-term cultures of primary B-lymphocytes. *In vivo*, intravenous injections of VPA were efficient for leukemia/lymphoma therapy in the sheep model leading to decreased lymphocyte numbers and tumor regression [6].

Based on these promising observations in an animal model, a similar approach was initiated in patients infected with HTLV-1 (Human T-lymphotropic Virus-1) [7,8]. It is estimated that 15 to 20 million persons live with HTLV-1 infection worldwide. HTLV-1 causes adult T-cell leukemia-lymphoma (ATLL), HTLV-1-associated myelopathy/tropical spastic paraparesis (HAM/TSP), uveitis and infective dermatitis [9]. There is still no satisfactory treatment of these different diseases [9]. Several HDAC inhibitors induce apoptosis of HTLV-1-infected cells and affect tumor growth [10,11,12]. In primary cells isolated from HAM/TSP patients, VPA has been shown to be proapototic, induce hyperacetylation and trigger viral expression [7]. Despite some initial fluctuations in the proviral loads, VPA treatment of HAM/TSP patients did not improve clinical symptoms after a two-year period [8]. Finally, VPA was used as consolidation therapy upon interruption of AZT-IFN treatment of ATLL patients [13]. VPA treatment might thus be of therapeutic interest for the treatment of HTLV-1 induced diseases. Therefore, we aimed at characterizing the mechanisms associated with VPA treatment in the BLV model.

## 2. Results and Discussion

### 2.1. Reduction of B Cell Counts upon VPA Treatment

**Figure 1:**
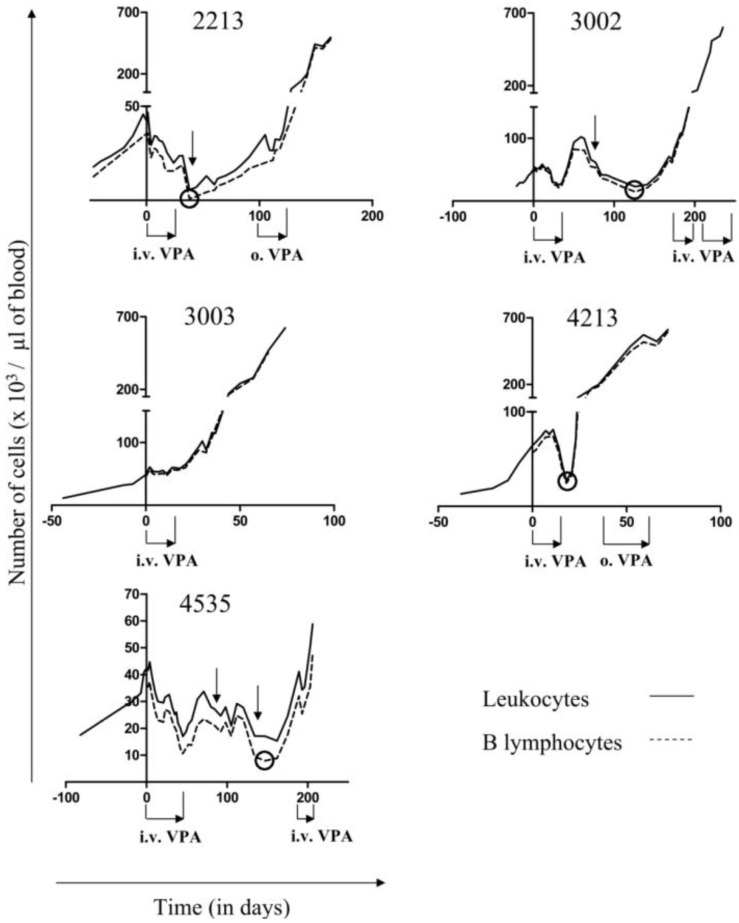
Valproate (VPA) treatment of bovine leukemia virus (BLV)-infected leukemic sheep VPA was administrated intravenously (10 g per injection, as indicated by: i.v. VPA) or orally to BLV-infected sheep (1 g per day as indicated by: o. VPA). The absolute number of leukocytes (black line) was determined with a hematological counter and the proportion of B lymphocytes was evaluated by flow cytometry using an anti-IgM antibody. The numbers of B-cells are represented by the dotted line. The black circles highlight the lower B cell counts observed within each sheep during treatment. The vertical black arrows show the decrease of the leukocyte and B cell counts occurring outside the VPA administration period.

Long-term disease progression was characterized in five BLV-infected sheep. At initiation of VPA treatment (day 0, Figure 1), all sheep were in the acute progressive phase of the disease as attested by the rapid increase in their leukocyte counts (Figure 1, black line), a majority being B-lymphocytes (Figure 1, dotted line). At this stage, leukemia is quickly lethal within a few days or weeks [2,6]. Indeed, in absence of treatment, the average time period between the initiation of the acute leukemic phase and the death of the animals is 46 days (with a standard deviation of 39 days) [6]. At day 0, numbers of B-cells were 35, 46, 46, 57 and 36 × 10^3^ cells per µL of blood in sheep # 2213, 3002, 3003, 4213 and 4535, respectively. The five leukemic BLV-infected sheep were treated by intravenous injections of 10g of VPA (Figure 1, i.v. VPA), as described previously [6]. The treatment provoked a direct reduction in the B-lymphocyte numbers in four sheep (*i.e.*, # 2213, 3002, 4213 and 4535), extending our previous observations [6], but not in animal # 3003 (Figure 1, dotted line). Interestingly, a time delay between VPA treatment and drop of lymphocyte counts could be observed (see vertical arrows on Figure 1).

### 2.2. Relapse and Onset of Chemoresistance against VPA Treatment

**Figure 2:**
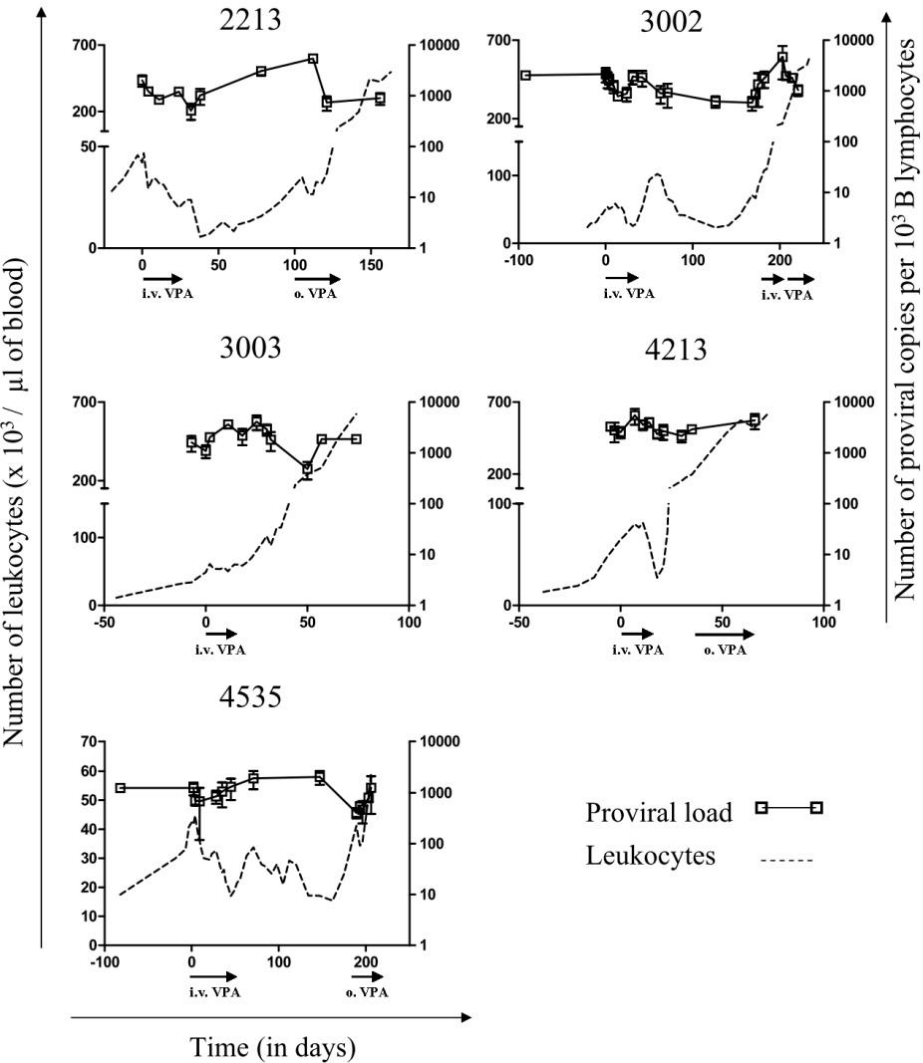
BLV proviral loads during VPA treatment and relapse: The proviral loads (empty squares), represented as numbers of viral copies per 10^3^ B-lymphocytes, were measured by real time PCR using genomic DNA isolated from sheep peripheral blood mononuclear cells (PBMCs). Data result from triplicate measurements (error bars represent means ± standard deviations). Absolute leukocyte numbers are indicated as reference (dotted line).

Upon interruption of treatment, the B-lymphocyte counts eventually increased in all sheep after different periods of time. During relapse, retreatment with VPA either orally (Figure 1, o. VPA) or intravenously (Figure 1, i.v. VPA) did not impede disease progression, leading to subsequent death. In other words, all sheep relapsed and evidently became unresponsive to VPA treatment. We next quantified the DNA proviral loads throughout VPA treatment and relapse using real time PCR (Figure 2, empty squares). In general, the relative proviral load (*i.e.*, the number of proviral copies per B-cell approached 1 (or 1.000 in 10^3^ B lymphocytes on the Y axis indicated on the right), with some sporadic fluctuations. This data thus confirms that leukemia in all five sheep was due to the expansion of BLV provirus carrying B lymphocytes. To conclude, it appeared that VPA treatment decreased the number of B-cells and the absolute PVL (*i.e.*, the number of proviral copies per mm^3^ of blood) but did not reduce the proportion of infected cells in the B-lymphocyte pool, even when close to normal cell counts were restored (at days 38, 126, 18 and 162 for sheep # 2213, # 3002, # 4213 and # 4535, respectively; see circles in Figure 1).

### 2.3. *Ex Vivo* Cell Cycle Distribution and Viral Expression during VPA Treatment and Relapse

**Figure 3:**
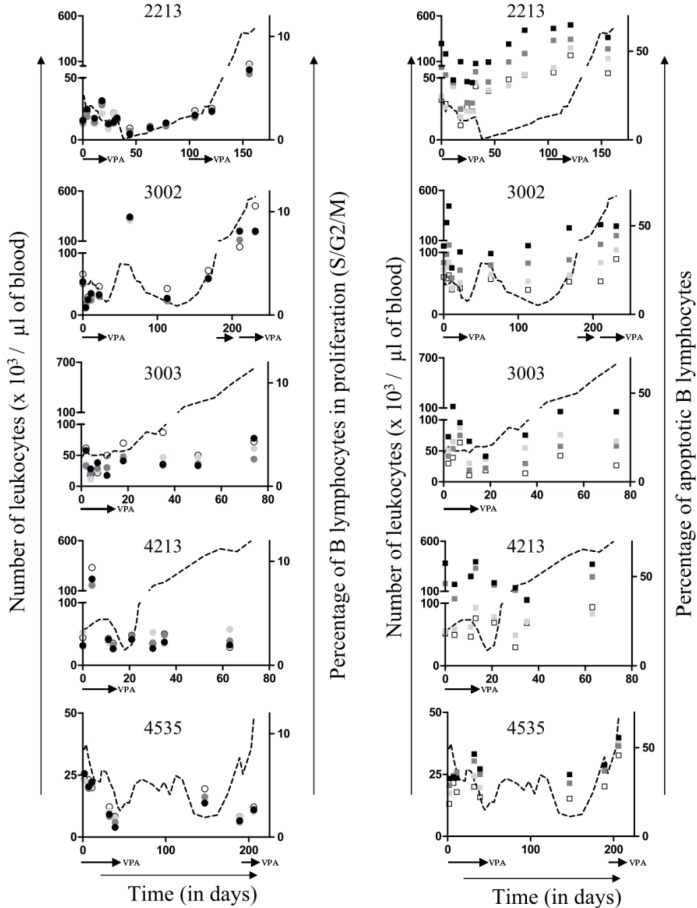
*Ex vivo* proliferation and apoptosis: PBMCs, isolated from BLV infected sheep during VPA treatment and relapse, were cultivated for 16 hours in absence or in presence of different concentrations of VPA (from 0, 1, 5 and 10 mM). The percentages of cells undergoing proliferation (left panels; ○, 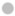, 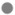
and 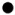 correspond to 0, 1, 5 and 10 mM of VPA, respectively) and apoptosis (right panels; □, 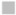, 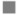 and 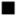 for 0, 1, 5 and 10 mM of VPA, respectively) were determined by flow cytometry. Absolute leukocyte numbers are indicated as reference (dotted line).

To further characterize the responsiveness of leukemic cells to VPA, peripheral blood mononuclear cells (PBMCs) were transiently cultivated *ex vivo* and analyzed by flow cytometry to assess apoptosis and cell proliferation. The levels of spontaneous apoptosis and the rates of cell proliferation fluctuated throughout treatment without apparent correlation neither with response efficiency nor with relapse (Figure 3, □ and ○). When added to the culture medium, VPA had only minor effects on the proportion of B-lymphocytes undergoing proliferation (Figure 3, left panels, 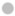, 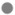 and 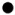 for respectively 1, 5 and 10mM of VPA) but clearly triggered apoptosis (Figure 3, right panels, 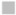, 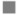 and 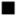 for respectively 1, 5 and 10mM of VPA). Importantly, this proapoptotic effect persisted throughout VPA treatment as well as during relapse.

**Figure 4:**
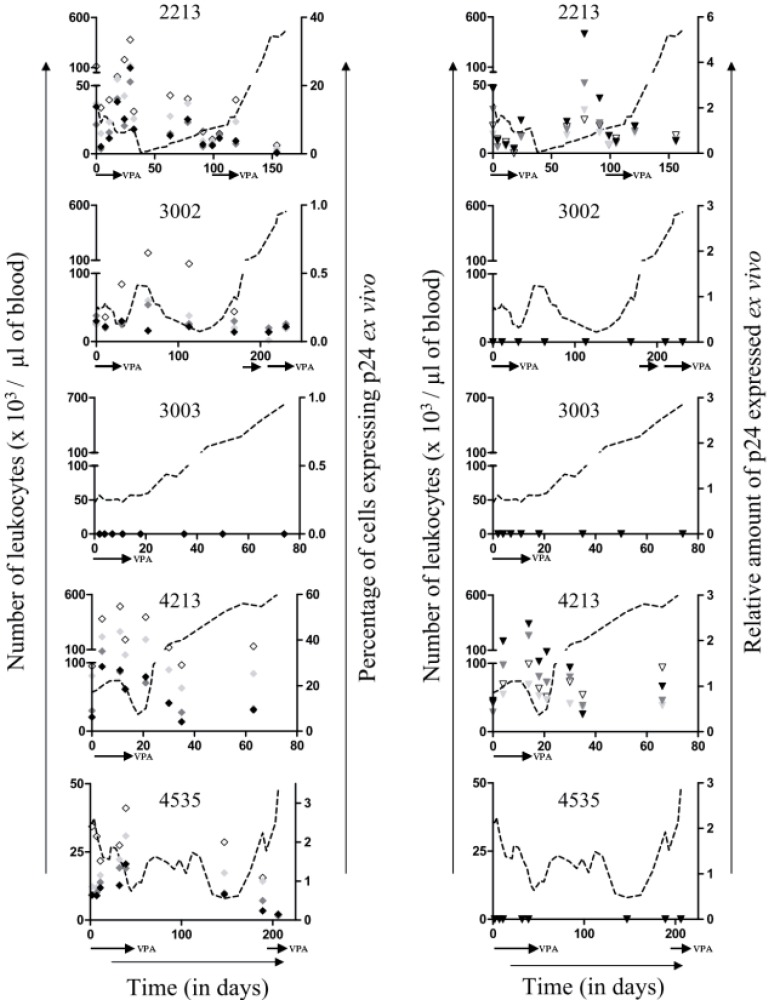
*Ex vivo* viral p24 expression: PBMCs, isolated from BLV infected sheep during VPA treatment and relapse, were cultivated during 16 hours in absence or in presence of different concentrations of VPA (from 0, 1, 5 and 10 mM). The percentages of cells expressing the viral protein p24 (left panels; ◊ for 0 mM, 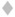, 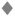, and 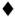 for respectively 1, 5 and 10 mM of VPA) were determined by flow cytometry. The amount of p24 in the supernatant (right panels; ∇ for 0 mM, 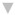, 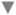 and 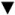 for respectively 1, 5 and 10 mM of VPA) was measured by ELISA. Data derives from optical densities measured in the linear range of a standard curve and are represented as relative amounts normalized for non-apoptotic B-cells. Absolute leukocyte numbers are indicated as reference (dotted line).

To evaluate the ability of infected B cells to spontaneously express viral proteins, PBMCs were transiently cultivated *ex vivo* and analyzed for expression of p24, the major core antigen of the viral particle. Two types of complementary techniques were used in parallel: flow cytometry to determine the percentage of cells containing p24 protein (Figure 4, left panels, ◊ ) and ELISA to quantify the relative amount expressed in the cell culture supernatant (Figure 4, right panels, ∇ ). It appeared that high levels of spontaneous p24 expression were measured in PBMC cultures from two sheep # 2213 and # 4213. Two other animals (# 3002 and # 4535) yielded low (*i.e.*, below 3%) but detectable percentages of p24-positive cells, the relative amount of p24 being below the sensitivity of the ELISA test (right panels). Finally, PBMCs isolated from one sheep (# 3003) were completely negative for p24 expression. There is thus a large disparity in p24 *ex vivo* expression among the five sheep. When PBMCs were cultivated *ex vivo* in the presence of VPA, the percentages of p24-positive cells decreased (Figure 4, left panels, 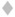, 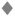, and 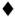 for respectively 1, 5 and 10mM of VPA), most likely due to the proapototic effect of VPA. In contrast, the relative amount of p24 antigen increased most of the time (Figure 4, right panels, 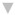, 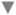 and 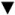 for respectively 1, 5 and 10mM of VPA) confirming previous observations [6]. Because apoptotic cells do not express viral proteins [2], the p24 burst is due to an increase of proviral expression.

### 2.4. Tumor Clone Replacement in Two out of Five BLV-Infected Sheep

**Figure 5:**
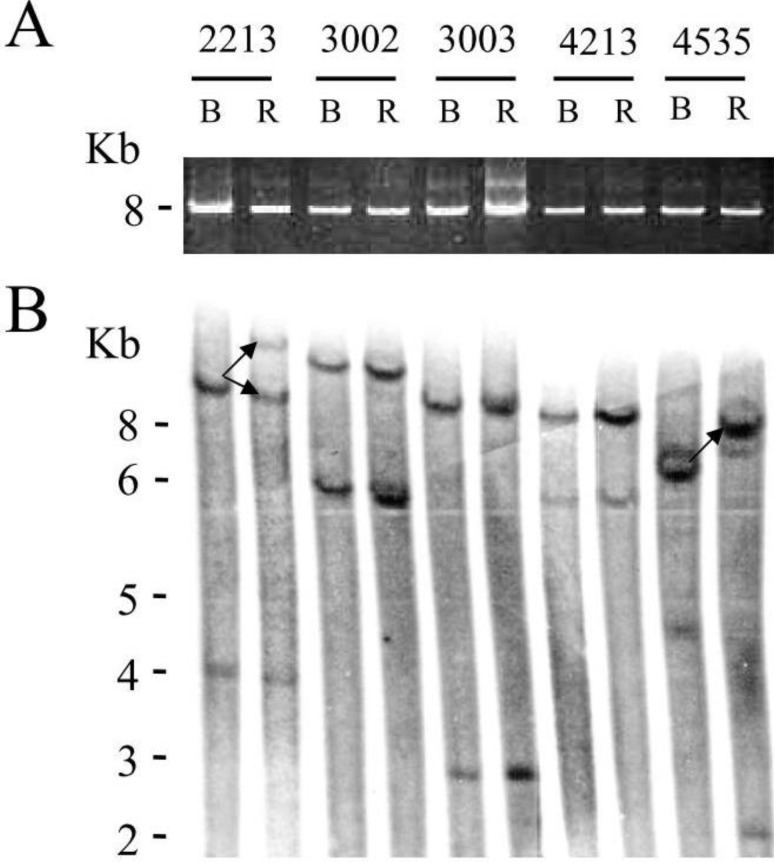
BLV proviral integrity and integration sites during VPA treatment and relapse: A. DNA was extracted from BLV-infected sheep PBMCs (2213, 3002, 3003, 4213, 4535) isolated just before VPA treatment at day 0 (B) and at the end of the relapse period (R). The full length proviral sequence was amplified by PCR using primers located in the 5' and 3' LTRs. PCR amplification products were migrated onto an agarose gel. The molecular weight of the amplicon is indicated in kilobases (8 Kb). B. Southern blot hybridization using a BLV probe of genomic DNA digested with EcoRI. The molecular weight markers (in Kb) are indicated on the left. Lanes correspond to those of panel A.

To evaluate proviral integrity, the full-length BLV genome was amplified by PCR using primers located in the 5' and 3' LTRs. It appeared that all sheep contained full length BLV proviruses before treatment (Figure 5A, “B”), as well as during relapse (“R”). To assess the clonality of viral integration, cellular DNA was digested with restriction enzyme EcoRI, which cuts the provirus sequence once and is analyzed by Southern blot using a BLV probe. The hybridization profile was altered during relapse in sheep 2213 and 4535 (Figure 5B, see arrows) indicating that the infected clones expanding before treatment and during relapse were different.

### 2.5. Treatment of VPA-Unresponsive Animals with Other HDAC Inhibitors

**Figure 6:**
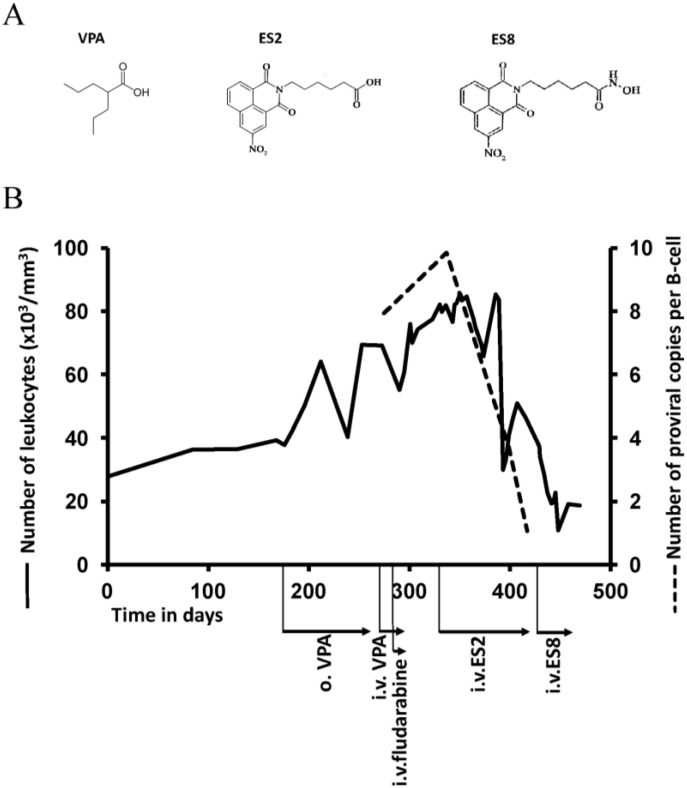
Treatment with newly developed HDAC inhibitors: (**A**) Chemical formula of VPA and 2 newly developed HDAC inhibitors; (**B**) Sheep #4270 was successively treated with VPA, Fludarabine, ES2 and ES8 (two newly developed HDAC inhibitors). VPA was mixed with food and administrated at a rate of 1 g/day during 48 days and at 3 g/day during 38 days to the BLV-infected sheep. Ten grams of VPA were also injected intravenously into the jugular vein alone (four times) or in combination with 50 mg of fludarabine (three times) spread over a period of 17 days. Increasing concentrations of ES2 (from 0.1 mg to 7 g, 24 injections) and ES8 (from 1.1mg to 56mg, 9 injections) were given intravenously.

Treatment of BLV-infected sheep #4270 was initiated at the leukemic phase of the disease (37.7 × 10^3^ cells/μL at day 176) with VPA given orally. The aim was to test whether or not this type of treatment could decrease the B-cell counts. This animal received serial oral administrations of VPA (1 g/day) (Figure 6B). Due to leukemia progression, 3 g of VPA was given daily. Since this increased dose was also inefficient, the sheep received serial i.v. injections of 10 g VPA (7 doses) and of fludarabine (50 mg, 3 doses), a nucleoside analog active in chronic lymphocytic leukemia. These treatments did not impair disease progression leading to very high blood cell counts (80 × 10^3^ cells/μL).

Facing unresponsiveness to VPA, our aim was to overcome chemoresistance and identify an efficient novel treatment of BLV-induced leukemia. Since VPA is a relatively weak HDAC inhibitor active only in the millimolar range, two improved compounds (ES2 and ES8) were developed (Boittiaux *et al.*, in preparation). ES2 shares the carboxyl HDAC inhibitory arm with VPA (Figure 6A). In ES8, the carboxyl is replaced by a hydroxamic acid which more potently interacts with the zinc ion located in the HDAC catalytic pocket. These inhibitors were tested for their ability to treat leukemia of animal #4270. Intravenous injections of ES2 transiently reduced cell counts but yielded again chemoresistance (Figure 6B). Further treatment with ES8 induced almost complete clearance of leukemia; B cell counts reaching levels close to normal and proviral loads decreasing dramatically (Figure 6B, dotted line). The animal died later on from causes unrelated to leukemia (*i.e.*, enterotoxemia). Unfortunately, treatment of 3 other leukemic animals with ES8 was inefficient (data not shown).

This pilot experiment thus shows that improved HDAC inhibitors can overcome multiple chemoresistances to VPA and fludarabine in 1 out of 4 leukemic sheep.

### 2.6. Discussion

We previously provided evidence that VPA activates BLV gene expression in transient transfection experiments and in short-term cultures of primary B-lymphocytes [6]. *In vivo*, VPA injections into sheep efficiently decreased the numbers of leukemic cells in the peripheral blood and led to tumor regression. Such VPA treatment does not impact T cell count (CD4, CD8 and γδ) in leukemic sheep, nor modify total leukocyte and B cell count in uninfected sheep [6]. In the present study, we describe the long term fate of five BLV-infected sheep upon interruption of VPA treatment. Different responses to treatment were observed: none (# 3003), partial (# 3002 and 4213) and almost complete (*i.e.*, with leukemic cells counts below 10,000 per µL of blood, # 2213 at day 38 and # 4535 at days 45 and 147) (Figure 1). In any of the treated animals, sequential intravenous injections of VPA led to complete clearance of all provirus carrying cells in the peripheral blood (Figure 2). Amazingly, a delay can occur between treatment with VPA and the decrease of the B-cell numbers as observed in sheep # 2213, 3002 and 4535 at days 19–22, 60–125 and 70–160, respectively (Figure 1, vertical arrows). Since the half-life of VPA in the plasma is about 16–17 h, it thus appears that cells may disappear long after having been in contact with the HDAC inhibitor. In fact, we already observed this type of phenomenon in our previous study [6]. In presence of a strong antiviral immune response, it is unlikely that cells expressing viral proteins would persist, even transiently, in these BLV-infected sheep. Therefore, we speculate that a second step is required for viral expression and subsequent cell destruction such as for example cell migration to the lymphoid tissues, antigenic dependent T-cell activation or induction of cell proliferation [2,5]. Further experiments will be required to gain insight into the mechanisms of VPA therapy. However, we think that induction of viral expression is a key parameter of this process because sheep # 3003, which was the only one to be unresponsive to VPA, did not contain any detectable cell that spontaneously expressed p24 viral protein. In addition, there is also a trend towards a reduction in viral expression during relapse of all sheep, when the leukemic cells accumulate exponentially. Importantly, we recently demonstrated that transient stimulation of viral expression *ex vivo* correlates with faster disappearance of B cells *in vivo* [14]. Although this model is still hypothetical, we propose that VPA activates viral expression in the silent pool of provirus carrying cells and permits their subsequent destruction by the host’s immune response. Therefore, mechanisms impairing viral expression such as proviral deletions or mutations and/or DNA methylation [15] might interfere with VPA efficacy. To achieve complete remission, it may therefore be required to extend the duration of VPA treatment, modify its mode of administration and/or combine with other epigenetic modulators (e.g., a DNMT inhibitor).

Although we favor an anti-leukemic effect based on transient induction of viral expression and subsequent destruction by the immune response, VPA is known to affect many other cellular mechanisms, including cell cycle arrest, apoptosis, differentiation, and angiogenesis [16,17]. VPA also potently induces lethal reactive oxygen species and reactivates cell death receptor pathways (TRAIL and CD95L/FasL) [18,19,20]. Consistently, our data of Figure 3 shows that VPA is proapoptotic in cell culture. However there is no direct correlation between VPA proapoptotic effect *ex vivo* and reduction of leukemic cells *in vivo*. To directly address this question, kinetic parameters (*i.e.*, proliferation and apoptosis) could be determined using intravenous injections of bromodeoxyuridine or carboxyfluorescein succinimidyl ester (CFSE) [21,22]. However, a mechanism based on direct induction of apoptosis by VPA does not fit with the time delay observed between its administration and reduction in leukemic cell numbers (arrows on Figure 1), further supporting our hypothetical “virus activation/immune destruction” model. In this context, the replacement of tumor clones, as attested by a modification of the proviral integration site (# 2213 and # 4535, Figure 5), also argues in favor of a selection process directed towards defined cell populations rather than via a general proapoptotic effect of VPA.

An important overall conclusion of this report is that all five sheep relapsed after different time periods post-treatment. During relapse of leukemia, we were unable to successfully constrict disease progression. As outlined above, inefficacy of VPA might be due to the lack of viral expression and/or impairment of immune response at final stages of leukemia. Resistance to VPA treatment may also be the consequence of VPA clearance. Conjugation with glucuronic acid followed by renal excretion is indeed the major metabolic route for VPA clearance [23,24,25]. However, determination of VPA plasmatic concentrations during relapse did not support this type of resistance process (data not shown). Other potentially contributing mechanisms include intracellular drug trafficking, alterations in drug-target interaction, metabolic changes (e.g., tolerance to stress conditions), escape from checkpoint control or defects in apoptotic pathways [26,27]. In particular, membrane transporters play important roles in reducing intracellular drug concentrations in tumor cells. ABC (ATP-binding cassette) transporters are frequently associated with decreased cellular accumulation of anticancer drugs and multidrug resistance of tumors [28]. However, efflux of VPA was reported to be independent of MRP-1/2 (multidrug resistance proteins) and P-glycoprotein [29,30,31].

Whatever the mechanism which remains to be further characterized, our report has important conclusions for the ongoing trials evaluating HDAC inhibitors in HTLV-1 therapy. In HAM/TSP, VPA treatment also appeared inefficient in permanently reducing the proviral loads after 2 years [8], likely due to similar mechanisms. Perhaps the combination of VPA with an anti-retroviral agent (AZT) could achieve this goal in human as described in STLV-1 infected *P. papio* [32]. Alternatively, improved HDAC inhibitors (Figure 6) may also be useful, although increased potency may correlate with exacerbated toxicity. In ATL, the overall median survival is about one year in its most aggressive forms (reviewed in [33]). Encouraging results were obtained in patients undergoing allogeneic stem cell transplantation but this procedure requires available donors and faces the problem of graft versus host disease [34]. Recently, a meta-analysis showed that ATL treatment with AZT and IFN induces high response rates and complete remissions and prolongs survival in acute, chronic and smoldering ATL, but not in lymphoma [35]. Unfortunately, many patients eventually relapsed, suggesting that AZT+IFN could not cure ATL and that new therapeutic approaches are still needed [36]. In this context, recent data from a small scale study indicated that VPA could be used as consolidation therapy after AZT+IFN [13]. Conclusions from our present report may be informative for further investigations of this approach.

## 3. Experimental

### 3.1. Experimental Design of VPA Treatment

Five sheep (# 2213, 3002, 3003, 4213, 4535) were maintained under restricted conditions (BSL-2) at the Study Center of Animal Productions (Gembloux, Belgium). Sheep were experimentally infected with a BLV wild-type cloned provirus (strain 344), as previously described [37]. At regular intervals of time, total leukocyte counts were determined by using an automated cell counter MS 4–5 vet (Mellet Schloesing Laboratories). To determine the percentages of B lymphocytes, PBMCs were labeled with an anti-IgM monoclonal antibody (clone 1H4), in association with rat anti-mouse IgG1 phycoerythrin, and then analyzed by flow cytometry (Becton Dickinson). Ten thousand events were collected for each sample, and data was analyzed with the Cellquest software (Becton Dickinson Immunocytometry Systems). Leukemic sheep (# 2213, 3002, 3003, 4213, 4535) received intravenous injections of VPA (respectively 9, 40, 9, 9 and 32 doses of 10 g each in 30 mL of sterile NaCl 0.9%) spread over several weeks, as indicated in the legend of Figure 1 (i.v. VPA). This amount of VPA thus corresponds to a daily dose of approximately 80 mg/kg/day, up to 250 mg/kg having been tested in sheep [24]. In animal # 3002, we had to interrupt VPA i.v. injections for a few days at the time of relapse because of a side effect (fainting). Since intravenous injections induce a transient but potentially harmful peak of plasmatic VPA concentration [6], we provided VPA orally, as for epilepsy therapy in humans. Sheep # 2213 and 4213 received oral VPA administrations (23 doses of 1 g per day each mixed with food) spread over several days, as indicated in the legend of Figure 1 (o. VPA).

### 3.2. Determination of the Proviral Loads

Venous blood was collected by jugular venipuncture and mixed with 0.3% w/v of EDTA used as an anticoagulant. 2 × 10^6^ peripheral blood mononuclear cells (PBMCs) were lysed in a buffer containing 100 mM Tris HCl pH 8, 150 mM NaCl, 10 mM EDTA and 0.5% SDS and digested overnight with RNAse A (0.1 mg/mL) and proteinase K (0.2 mg/mL). The cellular DNA was then purified by a phenol-chloroform (1:1) extraction and by ethanol precipitation. One hundred nanograms of genomic DNA were used for real-time PCR amplification of BLV proviral sequences as described previously [38,39]. Briefly, a segment corresponding to the pol gene (nucleotide coordinates 3,994–4,060 according to the BLV GenBank accession number K02120) was amplified with a pair of primers (5'-GAAACTCCAGAGCAATGGCATAA-3' and 5'-GGTTCGGCCATCGAGACA-3' at 900 nM final concentration) and revealed with a minor groove binder fluorescent probe (250 nM of 6FAM-CTCACCCACTGCAAC-MGB) using the TaqMan PCR universal master mix on a GeneAmp 5700 apparatus (Applied Biosystems). A standard curve was generated after amplification of defined proviral copy numbers (from 1 to 10^7^ of plasmid pBLV344) diluted in 100 ng of control genomic DNA. To correct for differences in DNA concentrations the 18S ribosomal DNA was quantified in parallel using probe 6FAM-CATGCCGACGGGCGCTGA-MGB and primers 5'-TTGGATAACTGTGGTAATTCTAGAAGCTAA-3’ and 5’-CGGGTTGGTTTTGATCTGATAAAT-3'.

### 3.3. PCR Amplification of the BLV Provirus

The full length proviral sequence was amplified with primers BLV3'RO (5'-CGCGCTTGTTTCCTGTCTTA-3'; position 232 to 251 according to the BLV GenBank accession number K02120) and BLV3'AS (5'-GACGTCT-CTGTCTGGTTTACGG-3'; position 8,245 to 8,224), using “Expand Long Range” according to the manufacturer’s recommendations (Roche). PCR reaction was performed with 250 ng of genomic cellular DNA template, 300nM of primer and 3% of DMSO with the following thermal cycling parameters: 96 °C 2 min, 10 × (96 °C 10 s, 62 °C 15 s, 68 °C 10 min), 20 × (96 °C 10 s, 62 °C 15 s, 68 °C 10 min + 20 s for each successive cycle), followed by a final elongation step of 7 minutes at 68 °C. PCR amplicons were finally resolved on a 0.8% agarose TAE (Tris acetate EDTA) gel.

### 3.4. Analysis of BLV Integration Sites by Southern Blot

Southern blot was performed with ten µg of genomic DNA digested at 37 °C during 3 hr with EcoRI restriction endonuclease. After ethanol precipitation, the digested DNAs were migrated on a 0.8% agarose gel, transferred onto a Hybond N+ membrane (GE Health), and hybridized with a BLV probe (Sac1 insert of plasmid pBLV344) labeled with α-32 P dCTP (Megaprime, GE Health).

### 3.5. Analysis of Apoptosis and Proliferation *ex Vivo* in Short Term Cultures of Peripheral Blood Mononuclear Cells

**Supplementary Figure 1:**
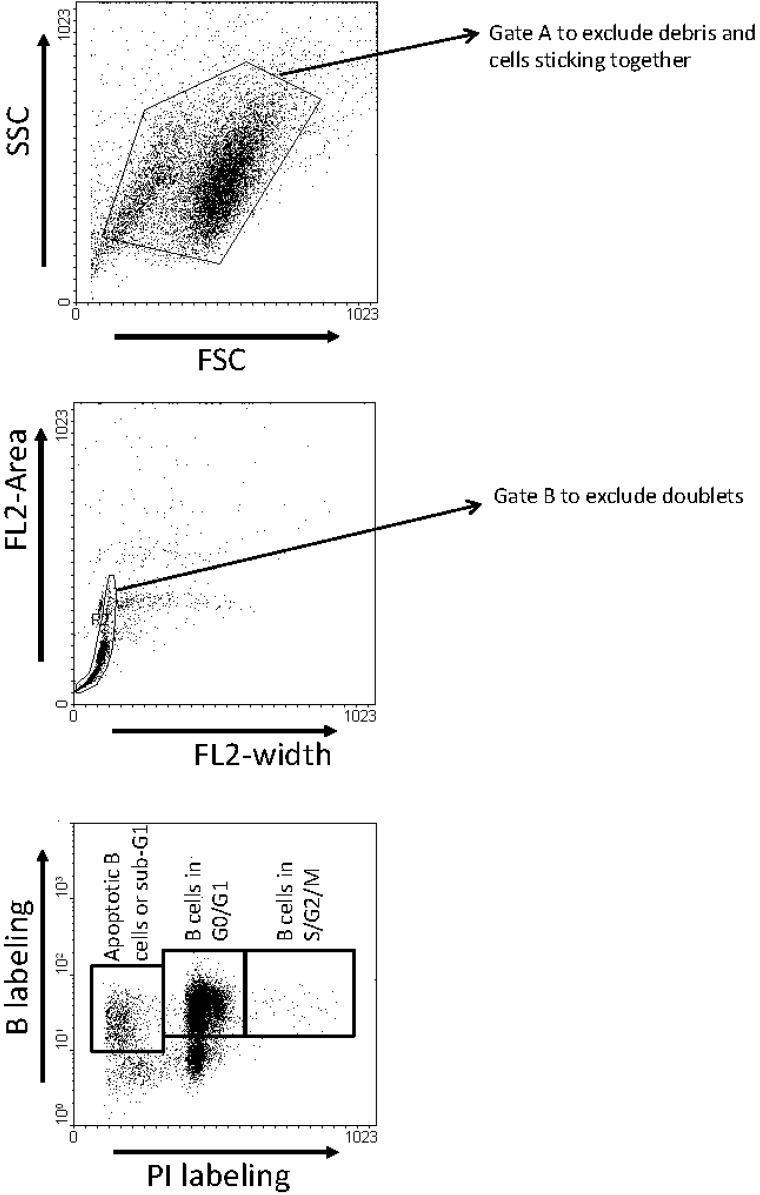
B-PI staining and gating technique: PBMCs were isolated from blood using a Percoll gradient. After B-cell labeling, ethanol fixation, and propidium iodide staining, the cells were analyzed by FACS. Cellular debris and clusters of cells were first excluded using Gate A (FSC vs SSC dotplot), then doublets were removed using Gate B (FL2-w vs FL2-A dotplot). A representative B-PI staining shows the 3 populations of B cells: apoptotic B-cells (or sub-G1), G0/G1 B-cells and proliferating B-cells (or S/G2/M).

PBMCs were separated by Percoll density gradient centrifugation (GE Health) and washed twice with phosphate-buffered saline (PBS)/0.075% EDTA and at least three times with PBS alone until complete clearance of platelets. After estimation of their viability by trypan blue dye exclusion, 2 × 10^6^ cells were cultivated during 16 hr at 37 °C in complete RPMI 1640 medium (e.g., supplemented with 10% FCS, 2 mM L-glutamine, 100 U of penicillin, 100 µg of streptomycin per mL; Sigma Aldrich) in the presence or the absence of different concentrations of VPA (Sigma Aldrich). After culture, PBMCs were collected, washed twice in PBS containing 10% FCS and incubated for 30 min at 4 °C in the presence of the monoclonal antibody 1H4 recognizing surface immunoglobulin M as a marker for B lymphocytes. Cells were then labeled with FITC-conjugated F(ab’)2 fragments of rabbit anti-mouse immunoglobulins (Dako) and fixed with 70% ethanol at −20 °C for at least 1 hr. After two final washes, the cells were treated with RNase A (50 µg/mL) (Sigma Aldrich) for 30 min at 37 °C, incubated for 5 min at room temperature in the presence of 20 µg per mL of propidium iodide (PI) (Sigma Aldrich) and analyzed by flow cytometry. Doublets were excluded from the analysis by using the (FL2a/FL2h/FL2w) gating method and cells staining in sub-G1 were considered to be apoptotic. Supplementary Figure 1 shows a representative B-PI staining and the gating technique.

### 3.6. Analysis of Viral Expression *ex Vivo* by Flow Cytometry and ELISA

To determine the proportion of cells expressing the p24 protein, sheep PBMCs were cultivated during 16 hr, fixed and permeabilized using DAKO IntraStain Reagent A and 1x Becton Dickinson Permeabilizing solution 2. Intracellular detection of p24 was performed by sequential incubation with 4'G9 monoclonal antibody and a rat anti-mouse IgG1 phycoerythrin conjugate (Becton Dickinson) for 30 min at 4 °C. Ten thousand events per sample were collected by flow cytometry and analyzed with the Cellquest software. To assess viral expression, culture supernatants were separated from PBMCs by centrifugation (10 min at 1,500 *g*) and analyzed for p24 protein amount using an enzyme-linked immunosorbent assay (ELISA) procedure. For this purpose, 96-well microtiter plates (Maxisorb immunoplate; Nunc) were coated with 4'G9 monoclonal antibody (300 ng in PBS per well) for 4 hr at room temperature. The plates were washed three times with PBS-Tween 80 (0.2%), and serial threefold dilutions of supernatants were added to the wells in presence of bovine serum albumin (0.67%) and Tween 80 (1.33%). After overnight incubation at 4 °C and three washes, the presence of the p24 major capsid antigen was revealed using two monoclonal antibodies (2'C1 and 4'F5) conjugated with horseradish peroxidase.

## 4. Conclusions

In summary, our data shows that (i) BLV-infected leukemic sheep relapse upon interruption of VPA therapy and may become refractory to further treatment; (ii) B lymphocytes isolated throughout treatment and relapse remain responsive to VPA-induced apoptosis in cell culture; (iii) B cell proliferation is marginally affected by VPA; (iv) *ex vivo* viral expression becomes nearly undetectable at the end of the relapse in four out of five sheep; (v) a new tumoral clone arose during relapse in two out of five sheep, most likely revealing a selection process exerted by VPA *in vivo*, and (vi) improved HDAC inhibitors can overcome chemoresistance in one out of four sheep. These observations in the BLV model may be important to improve current therapies of HTLV-induced diseases.
